# Photoluminescence enhancement with all-dielectric coherent metasurfaces

**DOI:** 10.1515/nanoph-2021-0640

**Published:** 2021-12-22

**Authors:** Yu-Tsung Lin, Amir Hassanfiroozi, Wei-Rou Jiang, Mei-Yi Liao, Wen-Jen Lee, Pin Chieh Wu

**Affiliations:** Department of Photonics, National Cheng Kung University, Tainan 70101, Taiwan, ROC; Department of Applied Chemistry, National Pingtung University, Pingtung 90003, Taiwan, ROC; Department of Applied Physics, National Pingtung University, Pingtung 90003, Taiwan, ROC

**Keywords:** array size effect, coherent collective resonance, Mie resonance, photoluminescence enhancement, TiO_2_ metasurface

## Abstract

Mie resonances have recently attracted much attention in research on dielectric metasurfaces, owning to their enriched multipole resonances, negligible optical loss, and efficient light emitter integration. Although there is a rapid advancement in this field, some fundamental developments are still required to provide a simpler and more versatile paradigm for photoluminescence (PL) control. In this work, we proposed that an all-dielectric coherent metasurface can engineer the PL response by tuning the array size. Such PL manipulation is attributed to the collective Mie resonances that mediate the inter-unit interactions between unit elements and alter the PL intensity. Metasurfaces with different chip sizes are utilized to explore the array size effect on the collective Mie resonances, field enhancement, and Q-factor in TiO_2_ metasurfaces. Incorporating the all-dielectric coherent metasurface with fluorescent photon emitters, we performed the dependence of PL enhancement on array size, which achieves an enhancement factor of ∼10 at the central area of a 90 × 90 μm^2^ TiO_2_ metasurface array. These findings provide an additional degree of freedom to engineer the near-field confinement and enhancement, allowing one to manipulate incoherent photon emission and tune light–matter interaction at the nanoscale.

## 1 Introduction

Photoluminescence (PL) has been intensively utilized in versatile fields such as biological imaging and sensing [1], [2], [3], [4], nanolaser [5], [6], [7], and quantum technology [8, 9]. Of particular interest has been the scenario that the PL intensity can be significantly enhanced when the photon emitters (quantum dots, fluorescence dye, etc.) are either coupling to an optical cavity [10] or located in the vicinity of plasmonic nanostructures [11, 12]. Since most physical parameters of metallic nanostructures like geometric sizes and shapes can strongly influence the photon–plasmon interaction, the realization of PL enhancement via subwavelength plasmonic fields has attracted increased interest over the past decade. However, plasmonic nanostructures often suffer from high dissipative Joule losses while resonating, which degrades the device performance and impede real-world applications. A dramatic decrease of quantum yield can also occur if the spatial distance between photon emitters and metallic structure is not well designed [13, 14], bringing about the quenching of PL. As a consequence, dielectric nanostructures and metasurfaces become an attractive topic of intense investigation for PL manipulation.

All-dielectric metasurfaces have been explored widely owing to their negligible dissipative loss and capability for efficient light manipulation [15]. For example, low-profile optical components with metasurface optics such as high-performance metalens [16], [17], [18], holographic imaging [19], [20], [21], and entangled photon generator [22], [23], [24] have been demonstrated with record-high efficiency. Possessing a relatively high refractive index, both in-plane and out-of-plane displacement current oscillations can be substantially induced in dielectric metasurfaces. In such a way, not only the Mie resonances but also toroidal multipolar modes can be possibly excited in an isolated structure [25]. The coexistence of electric and magnetic Mie resonances in dielectric Huygens’ metasurfaces gives rise to diverse applications, including control of far-field radiation patterns [26], nonlinearity enhancement [27], highly-transmissive metasurfaces [28], and optical sensing [29, 30]. The Mie, as well as toroidal resonant modes and interactions among them further offer a new channel to engineering electromagnetic field distributions in the near-field region, which enables modulation of PL characteristics when coupling to emitted photons [25, 31]. However, in comparison with their metallic counterparts, the coupling efficiency between photon emitters and dielectric metasurfaces is much weaker. It is because that the dielectric nanoresonator often acts as an open nanocavity, making high field confinement inside the metasurface [32, 33]. Thus, it is highly desired to have the mode fields spatially overlapping with the photon emitter for yielding an enhanced coupling efficiency and PL intensity boost. One potential solution to address the issue is to integrate the photon emitter inside the dielectric nanoresonators [33, 34]. However, the requirement of several top-down fabrication steps complicates the metasurface manufacturing and limits the applications. Another promising method is creating field leakage into the air gap between metasurface unit elements, which can be realized by introducing near-field coupling. It has been explored that a nearly 2π phase modulation can be achieved by tuning the lattice periodicity [35, 36], revealing that strong mode coupling can occur between neighbors in the Huygens’ metasurface.

**Figure 1:**
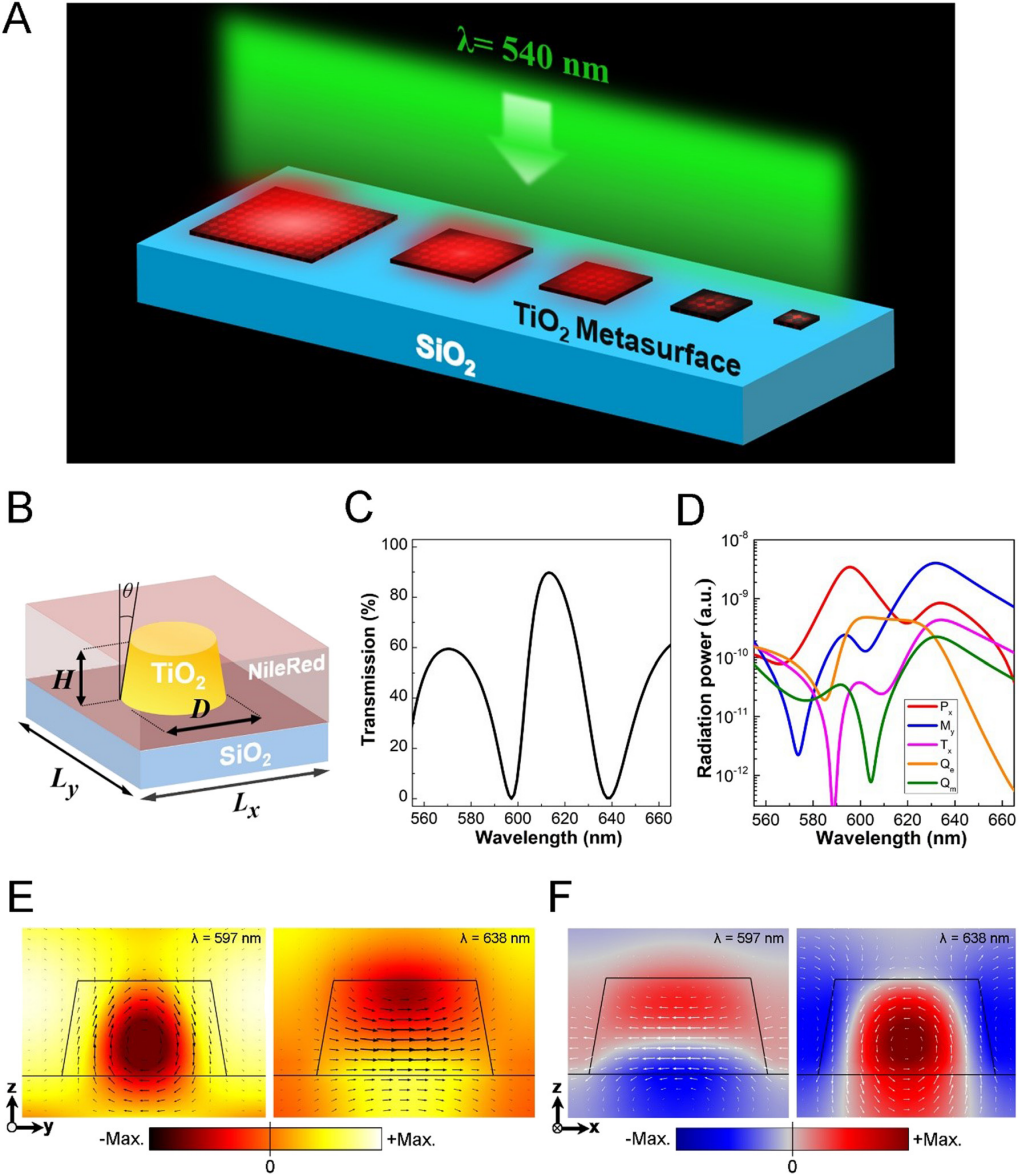
(A) A conceptual depiction of PL modification via all-dielectric metasurface array size. Due to the coherent resonance in the TiO_2_ metasurfaces, the PL intensity becomes higher as the array size increased. (B) Schematic illustration of the TiO_2_ dielectric metasurface unit standing on a SiO_2_ substrate. The diameter of the bottom of the nanofrustum *D* = 315 nm; the height *H* = 170 nm; the periodicity along *x*-direction *L*_*x*_ and *y*-direction *L*_*y*_ are 410 and 490 nm, respectively. *θ* represents the sidewall tapering angle that is fixed at 9° in this work. The dielectric metasurface is covered by a layer of fluorescent dye Nile red (NR) for emission control. (C) Simulated transmission spectra of the integrated TiO_2_-NR metasurface under an *x*-polarized illumination. (D) Calculated scattered power of the dielectric metasurface embedded in the NR for individual electromagnetic multipoles. *P*_i_: i component of electric dipole, *M*_i_: i component of magnetic dipole, *T*_i_: i component of toroidal dipole, *Q*_e_: electric quadrupole, *Q*_m_: magnetic quadrupole. Numerical (E) *x*-component of electric and (F) *y*-component of magnetic field distributions of the electric resonant mode (597 nm) and magnetic resonant mode (638 nm) of the TiO_2_ metasurface. Black arrows and white arrows respectively represent the magnetic field and the electric field.

In this study, we leverage the advances of coherent property in TiO_2_ Huygens’ metasurfaces for PL enhancement in the visible. In previous demonstrations of metasurfaces for phase modulation, it was inferred that the inter-unit coupling was essentially weak to be negligible, that is, the individual metasurface unit was regarded as an isolated nanoresonator at the design step. Here, benefitting from inter-unit interactions, we demonstrate that both the near-field enhancement and quality factor (Q-factor) of the all-dielectric Huygens’ metasurface turn to the coherent collective resonance in all unit elements. A weak resonance dominated by individual nanoresonators is merely caused by Mie scattering while aiming the coherent resonance can obtain utterly stronger resonance signals in the spectrum and hence provide abundant flexibility in emission modification. As a consequence, the finite size of the metasurface array can significantly influence the optical response of emitted photons, as schematically illustrated in Figure 1A. Based on the metasurface array size engineering, we numerically demonstrate and experimentally verify that the Q-factor, electric field intensity, and PL emission can be modulated. We argue that the field induced by a Huygens’ metasurface with finite array size is mostly confined at the center of the array and will be gradually diminished near the boundary. The light emission intensity may be directly linked to the collective oscillation underpinning the resonance. As a result, the PL enhancement becomes higher when the metasurface array size is increased. Combining the array-size-dependent resonance linewidth with a strong near-field boost, we realize a PL enhancement of ∼10 at a wavelength of 620 nm in a 90 × 90 μm^2^ TiO_2_ metasurface. Our works provide a facile alternative for tuning PL enhancement by the nanostructure array size in a simple symmetric design, and these may help understand the mechanism in the number of unit elements forming the coherent response for the control of light emission in dielectric metasurfaces.

## 2 Results and discussion

Figure 1B schematically illustrates the proposed dielectric metasurface composed of a TiO_2_ nanofrustum standing on a silica substrate. The periodicity along *x*-direction and *y*-direction are *L*_*x*_ = 410 nm and *L*_*y*_ = 490 nm, respectively. The nanofrustums are covered by a layer of Nile red (NR) which is utilized as the fluorescence emitter. The structural parameters are optimized to ensure that the Mie resonances of metasurface can occur when the NR is present. Figure 1C shows the simulated transmission spectrum of the TiO_2_ metasurface (infinite array) under an incidence of *x*-polarized light. As can be seen, the optical response is dominated by two resonances in the spectral range of 580–660 nm. According to the multipole decomposition calculation and analysis (see Figure 1D) [37], the resonance at 597 nm wavelength mainly results from the contribution of electric dipole along the *x*-direction (P_*x*_, red curve in Figure 1D). On the other hand, the resonance at 638 nm wavelength is characterized by magnetic dipole along the *y*-direction (*M*_*y*_, blue curve in Figure 1D), while the contribution from other electromagnetic multipoles like toroidal dipole (*T*_*x*_), electric quadrupole, and magnetic quadrupoles (*Q*_e_ and *Q*_m_) are relatively weak in the wavelength range of interest. The excitation of Mie resonances can also be evaluated from the electromagnetic intensity distribution on different cutting planes. As can be seen in Figure 1E and F, for the resonant mode at 597 nm, the circulating magnetic field profile along with a strong electric field enhancement gives rise to the excitation of electric resonance. The vortex electric field distribution and strong magnetic field enhancement support the excitation of magnetic resonance at 638 nm. Notice that since we considered the inevitably lateral erosion that often happens during the dry etching (refer to Figure S1 for more details), the TiO_2_ nanofrustums were modeled with a sidewall tapering angle of 9° in simulations (see Figure 1B, E, and F). Furthermore, we found that the resonant fields are concentrated not only inside the dielectric nanofrustum but the ambient space between metasurface units, especially for the electric resonance. The strong field enhancement in the air gap between nanofrustums reveals that the near-field inter-unit coupling indeed exists in the TiO_2_ Huygens’ metasurface. Because the transition rate of the fluorescent molecules is proportional to the electric field intensity at the nanoscale [38], such inter-unit interaction offers a channel to increase the spatial overlap as well as the coupling of emitters with the mode fields for photon emission boost. In the following sections, we will show that such inter-unit coupling leads to a coherent collective resonance (CCR) in the periodic dielectric metasurface. Thus, both the Q-factor of spectral features and the PL enhancement can be tailored via the finite size of metasurface array.

**Figure 2:**
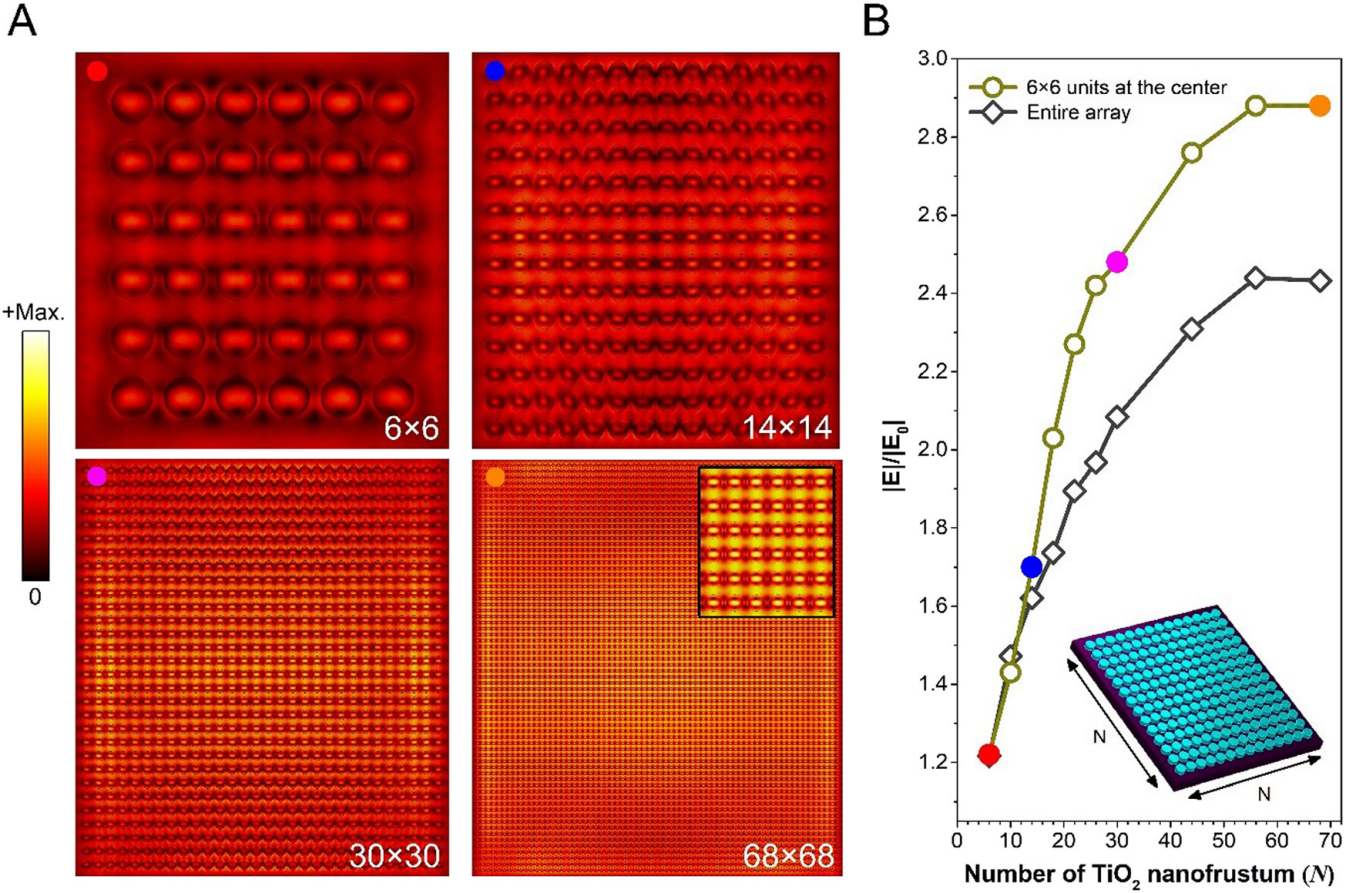
(A) Spatial electric field intensity distributions of four dielectric metasurface arrays in which each array consists of *N* × *N* TiO_2_ nanostructures. Inset in the 68 × 68 array shows a zoom-in image for clarity. The corresponding electric field enhancements at the center of each array are shown in (B). (B) Electric field enhancement versus the metasurface array size. The field enhancements are respectively carried out from a fixed area (6 × 6 nanostructures at the center of individual array) and the entire array for comparison. The electric intensity is normalized to the intensity of incident wave (*E*_0_). Inset: schematic of an *N* × *N* metasurface array.

Before exploring the effect of the array size on PL enhancement, we investigated the CCR in the TiO_2_ metasurface by simulating the electric field enhancement for various array sizes. Figure 2A shows the electric field intensity distribution at the electric resonance wavelength for metasurface arrays composed of *N* × *N* TiO_2_ nanofrustums (*N*: the number of units along the side of the metasurface array, as illustrated in the inset of Figure 2B). As shown in Figure 2A, the near-field inter-unit coupling mediated by the electric resonance is observed in all cases, leading to the realization of field enhancement between neighboring units. The field intensity becomes higher as the metasurface array size increases, revealing the coherent response in the TiO_2_ metasurface. In addition, we found that such collective resonance commences being intensified at the center of arrays especially for the interaction between TiO_2_ nanofrustums along *y*-direction (see inset of Figures 2A and S3). This is because the nanostructures at the edge of the metasurface array can experience very different field distribution so that the scattering from dipole radiation comes to dominate the response at the array boundaries [39]. As a consequence, the 6 × 6 array possesses nonuniform field distribution over the metasurface. For the other metasurfaces with larger array sizes, the innermost dielectric nanofrustums show greater electric field intensity than the nanostructures nearby the edge of the array. Such nonuniform field enhancement characterization turns into much more obvious for the 30 × 30 and 68 × 68 metasurface arrays. To demonstrate the dependency of the field intensity on the total number of TiO_2_ nanofrustums, we provide the electric field enhancement spectrum versus the metasurface array size. For a fair comparison, the field enhancements are carried out in a fixed area (6 × 6 units) at the center of each metasurface array (dark-yellow circles in Figure 2B). As can be seen in Figure 2B, the field enhancement for the centermost TiO_2_ nanofrustums rises drastically as the metasurface array size increases. Even very much nonuniform field enhancement occurs for large metasurface arrays, we can also obtain a similar tendency of field enhancement when considering the entire metasurface (refer to black diamonds in Figure 2B). These results indicate that the inter-unit interaction via electric resonance in TiO_2_ nanofrustums strongly depends on the size of the metasurface array, i.e., the total number of units engaged in the near-field coupling, which again affirms the coherent response in the proposed metasurface.

**Figure 3:**
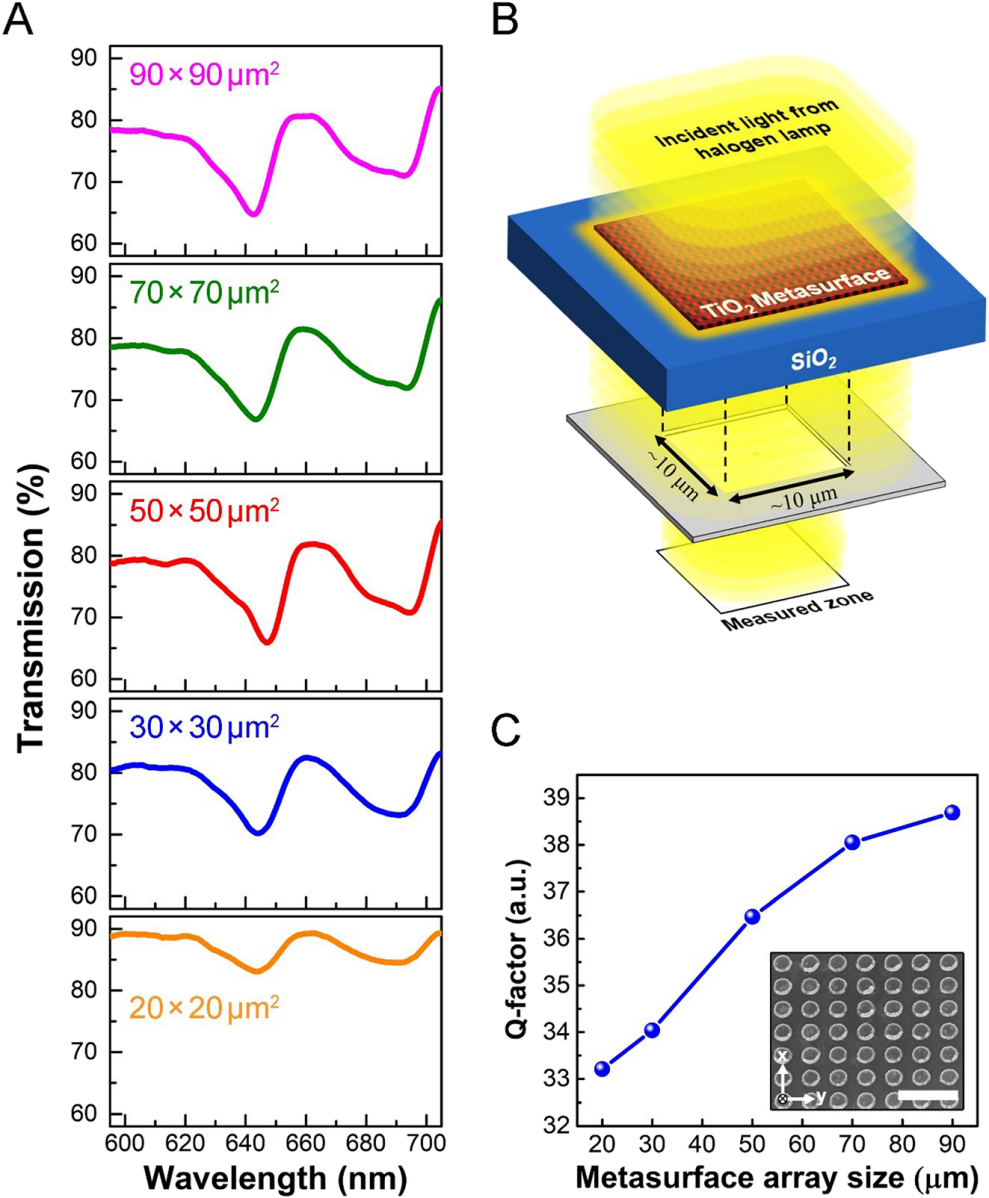
(A) Measured transmission spectra of the TiO_2_ metasurface arrays embedded in NR. (B) Schematic illustration for the optical measurement of the transmission spectrum. To characterize the transmission response at the central position of the individual metasurface array, a 10 × 10 μm metallic shutter gate is used. More details for the optical setup can be found in the Supplementary information. (C) Experimentally measured Q-factor of the electric resonant dip at a wavelength of ∼640 nm. Inset: SEM image of a fabricated 90 × 90 μm^2^ metasurface array sample. Scale bar: 1 μm.

To experimentally demonstrate the coherent electric resonance in the dielectric metasurfaces, we fabricated five TiO_2_ nanofrustum chips with array size varying from 20 × 20 μm^2^ to 90 × 90 μm^2^. Because the wavelength of Mie resonance is highly sensitive to the surrounding environment, to comprehensively study the spectral overlap of electric resonance and photon emission from NR, we directly characterize the transmission of fabricated TiO_2_ metasurfaces embedded in NR, as shown in Figure 3A. According to the results and discussions for Figure 2, the electric field enhancement at the center of the array is supposed to be much higher than the edges. Thus, the optical spectra shown in Figure 3A are collected through a fixed detection area of 10 × 10 μm^2^ at the center of metasurface arrays, as illustrated in Figure 3B. Here, the detection area is controlled by using a metallic shutter. More details of optical setups can be found in Figure S5. Overall, the features in measured spectra agree with the numerical simulations except for a red-shift of about 40 nm in resonant wavelengths. We attribute this difference in resonant wavelengths to the physical dimension discrepancy between numerical design and real samples. At the electric resonant wavelength of about 640 nm, the field intensity is highly concentrated in the collective inter-unit coupling in TiO_2_ nanofrustums that are coherently oscillating with the electric dipoles (see left image in Figure 1E and F). Thus, the metasurface array size plays a crucial role in the Q-factor in transmission. Figure 3C shows the extracted Q-factor as a function of metasurface array size. As shown in Figure 3C, the experimental Q-factors vary monotonically as the array size increased. The Q-factor is increased by ∼20% when the TiO_2_ metasurface array size enlarged from 20 × 20 μm^2^ to 90 × 90 μm^2^_._ The observation of a high Q-factor response with a larger number of TiO_2_ nanofrustums verifies the existence of a relatively low nonradiative loss as well as a strong inter-unit coupling between the nanoresonators. These results imply that an intensified CCR has been induced among the TiO_2_ metasurface, which is consistent with our numerical prediction (refer to Figure 2).

**Figure 4:**
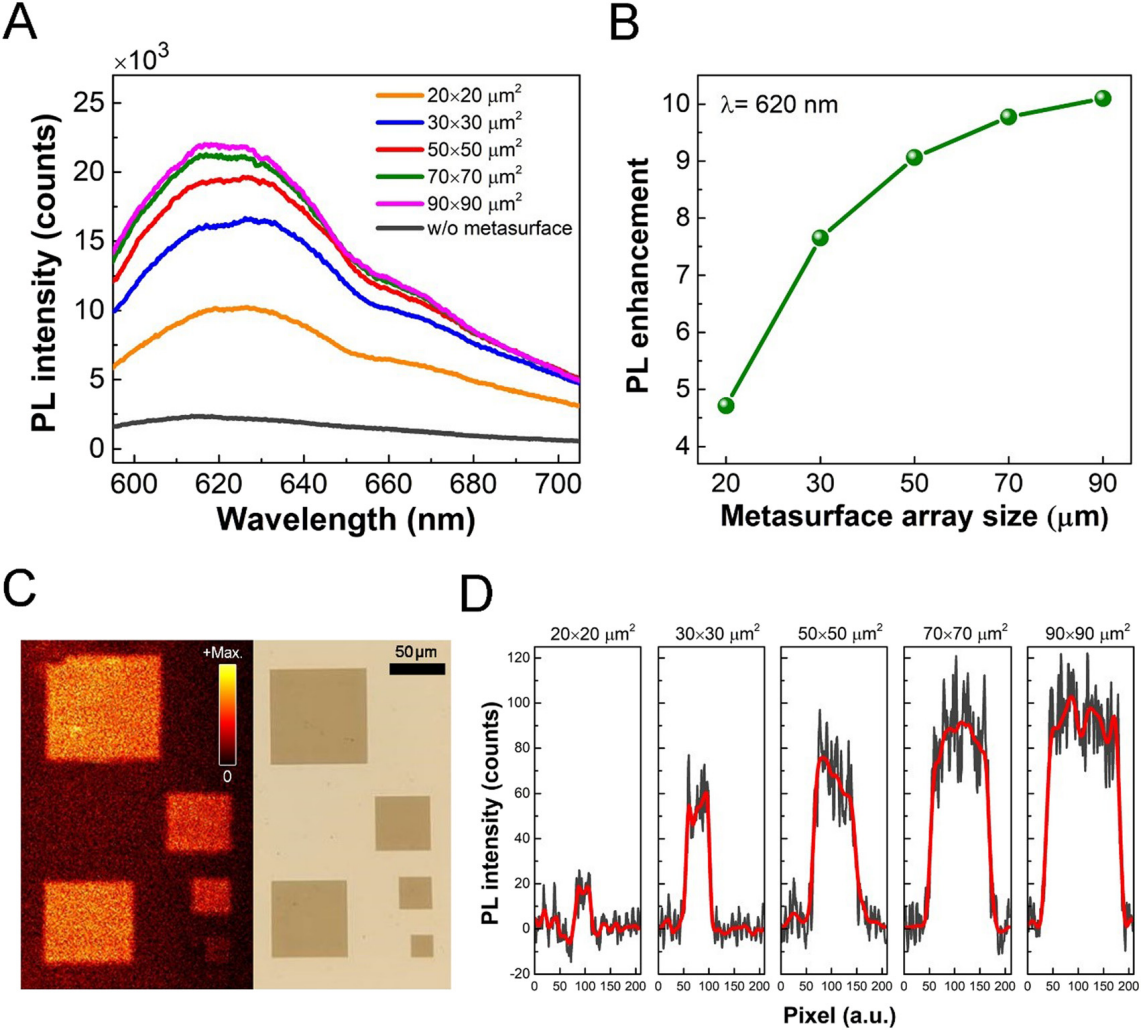
(A) Experimental PL intensity versus metasurface array sizes. The signal measured on the unpatterned area is plotted as a reference for comparison. (B) The PL enhancement at *λ* = 620 nm as a function of metasurface array size. (C) Spatial PL mapping (left) of the integrated TiO_2_-NR metasurfaces. The right image presents the corresponding microscopic image of the TiO_2_ metasurfaces. (D) Cross-sectional line-scanned PL intensity at the center of each array. Note that the results are deducted from individual background signals for clarity. Black curves: raw data. Red curves: smoothed curve. The excitation laser wavelength is 532 nm.

Based on the previous results and discussions, we conclude that a strong collective oscillation occurs over the large size of the TiO_2_ metasurface array along with strong electric dipolar field intensity at the center of the array. Next, we demonstrate the application of PL enhancement via the observed CCR in the TiO_2_ metasurface. Again, to focus on the influence of central TiO_2_ nanostructures on photon emission, the PL signal is collected with a fixed 10 × 10 μm^2^ area while all metasurfaces on the sample are optically excited. The use of the shutter gate for a fixed collection area is also helpful for evaluating the PL enhancement because the PL intensity highly depends on the NR volume. Figure 4A shows the PL intensity spectra modified with various metasurface array sizes. The PL signal of NR on an unpatterned substrate is plotted as a reference (black curve) for comparison. Thanks to the relatively broad photon emission response in NR, the resonant mode in TiO_2_ metasurface is still able to interact with the emitted photon even the PL emission peak doesn’t highly match with the electric resonant dip (refer to Figures 3 and 4A). As seen in Figure 4A, a drastic PL enhancement from the integrated TiO_2_-NR metasurfaces is observed over a weak background even in the smallest metasurface array. For all cases, the PL signal reaches the highest intensity at around 620 nm (which highly matches to the peak of the Purcell factor spectrum, see Figure S7) as being close to the center of NR emission. In addition, we find that the PL intensity increases rapidly for small metasurface arrays and starts to saturate when the metasurface array is greater than 70 × 70 μm^2^. To quantitatively analyze the PL enhancement, we provided the PL enhancement as a function of metasurface array size. The PL enhancement is defined by the ratio of the PL intensity on the patterned area at the center of the individual array to the value on the unpatterned area of the sample. The measured results in Figure 4B show that the PL enhancement factor is ∼4.6 for the 20 × 20 μm^2^ metasurface array and it reaches ∼10 when the array size is further increased to 90 × 90 μm^2^_._ These results imply that the CCR in TiO_2_ metasurface is indeed capable of coupling effectively with the NR photon emission. To further support our assertions, we perform spatial PL mapping measurements for all metasurface arrays (refer to Figure S6 for details of the optical setup). Instead of analyzing the PL enhancement by considering the contribution from a 10 × 10 μm^2^ area, the implementation of PL map enables us to investigate how the inter-unit coupling between TiO_2_ nanofrustums and array size influence the PL intensity with higher spatial resolution. The left image of Figure 4C shows the spatially mapped PL response of the TiO_2_-NR integrated metasurface sample. The PL map represents that the PL emission is relatively weak for smaller metasurface arrays, and the PL intensity increases with increasing the array size. To investigate the overall performance of the PL enhancement, we additionally examine the line-scanned PL intensity distribution extracted from the spatial map shown in the left image of Figure 4C. As seen in Figure 4D, the PL intensity presents a higher value over each metasurface than the background, confirming the capability of PL enhancement using CCR in the dielectric metasurface. Furthermore, we found that, while the PL intensity varies across the metasurface array because of the slightly different near-field coupling environment of the NR to the nanofrustums, the overall PL emission is boosted in the metasurface area. These results prove that the collective response among the array plays an important role in PL enhancement, which can be directly tuned by engineering the size of the coherent metasurface array.

## 3 Conclusions

In conclusion, we have performed the array-size-dependent PL enhancement via the CCR in all-dielectric TiO_2_ metasurfaces that feature a simple symmetric design for the unit element. The numerically calculated field profile and experimentally measured transmission spectra illustrate that the coherent all-dielectric metasurface exhibits stronger collective Mie resonance among the TiO_2_ nanofrustums as the array size increases. Via the spectrally-resolved PL measurement and spatially-resolved PL map, we have verified that the PL enhancement is highly dependent on the metasurface array size. In this study, a considerable PL enhancement of ∼4.6 is observed at the central area of a 20 × 20 μm^2^ metasurface array under a 540 nm excitation from a tungsten lamp, and the enhancement factor increases to ∼10 when the metasurface array size is enlarged to 90 × 90 μm^2^. The dependence between electric field enhancement, Q-factor in the transmission spectrum, and metasurface array size confirms that the collective inter-unit coupling of the Mie resonance contributes to the PL enhancement, particularly at the center of the metasurface array. According to the simulation results and the spatial PL mapping measurements, the scattering from the edges of the array is the most primary source of optical loss in the small metasurface chips, which leads to a suppression to the electric field intensity of collective resonance and hence to the PL enhancement. Such radiative loss at edges turns into a nearly negligible effect for the metasurface with larger chip size, resulting in a stagnation of PL intensity. Finally, we would like to point out that the Q-factor as well as the PL enhancement could be potentially further increased when other resonant modes with relatively low radiative loss are introduced. For example, the toroidal multipoles have been verified to exhibit stronger field confinement and much weaker far-field radiation loss in comparison with the electric and magnetic multipoles [37, 40, 41]. The collective coherent response has also been observed in toroidal metasurfaces [42]. Thus, we hypothesize that the performance of the array-size dependent PL response can be further improved when the toroidal mode is engaged with the coherent metasurfaces. The finding of array-size-dependent photon emission in coherent TiO_2_ metasurfaces provides a facile alternative for PL enhancement and manipulation. We anticipate that the results of this work hold a great promise for efficient light emission control at the nanoscale and advanced nanophotonics applications including bio-sensors and imaging, nanolaser, and quantum optics.

## 4 Methods

### 4.1 Simulation

The finite element method (COMSOL Multiphysics) is used for all numerical simulations. For the design of the TiO_2_ metasurface unit, including the calculation of transmission spectrum and scattered power spectrum, the periodic boundary condition is applied to both *x*-direction and *y*-direction. Tables S1 and S2 list the formulas for the calculation of individual electromagnetic multipoles and corresponding scattered power. The refractive index of SiO_2_, TiO_2_, and NR dissolved in polymethyl methacrylate (PMMA) are obtained from ellipsometry measurement (refer to Figure S4).

### 4.2 Fabrication

To fabricate the TiO_2_ metasurface, a 170-nm-thick TiO_2_ film was firstly deposited on a fused silica substrate by an atomic layer deposition system [43]. The thickness of the deposited thin film was confirmed by the step profiler. Then, the PMMA photoresist was spin-coated on the pre-prepared substrate and baked at 180 °C on a hot plate for 3 min. Subsequently, the photoresist was exposed to an e-beam direct writing system (Elionix ELS-7500) to define the nanopatterns, followed by a development process for 2 min with the PMMA developer (MIBK:IPA = 1:3). The proximity effect correction was considered during the exposure process. After the development process, 70-nm-thick chromium was deposited onto the sample by an e-beam evaporator. Subsequently, the lift-off method was employed with soaking the sample into the PG remover at 100 °C for 1 h. After ensuring the photoresist was completely removed, the sample was then etched by the inductively coupled plasma system through the usage of mixed gases of CHF_3_ and Ar in a ratio of 10 sccm to 30 sccm. Finally, the chromium protection layer was removed using the Cr-7 from TiO_2_ nanostructures. A high-precision FIB scanning electron microscope had used to confirm the depth of the nanostructure.

## Supplementary Material

Supplementary Material
