# Supplementary material for: Photoluminescence enhancement with all-dielectric coherent metasurfaces
Source: Nanophotonics. 2021 Dec 22;11(11):2701–9. doi: 10.1515/nanoph-2021-0640 (PMC11501412; doi:10.1515/nanoph-2021-0640)
Supplement: Supplementary file 1 — Supplementary Material [file j_nanoph-2021-0640_suppl_001.pdf]

Supporting Information for

Photoluminescence Enhancement with All-dielectric Coherent Metasurfaces

Yu-Tsung Lin[1], *Amir Hassanfiroozi*[1], *Wei-Rou Jiang*[1], *Mei-Yi Liao*[2], *Wen-Jen Lee*[3], *Pin Chieh Wu*[1,*]

[1]Department of Photonics, National Cheng Kung University, Tainan 70101, Taiwan

[2]Department of Applied Chemistry, National Pingtung University, Pingtung 90003, Taiwan

[3]Department of Applied Physics, National Pingtung University, Pingtung 90003, Taiwan

*E-mail: pcwu@gs.ncku.edu.tw

1. SEM images of the fabricated TiO_2 nanofrustums

Due to the lateral erosion during the ICP etching process, the nanostructures can possess a tapering angle at the sidewall. Indeed, such tapering angle can be controlled via the DC power. For example, in Fig. S1C, a larger tapering angle of ~15˚ was realized by using a DC power of 80 W. When the power is increased to 250 W (the parameter we used for the fabrication of all samples presented in this work), the tapering angle becomes less acute (~ 9.2˚).

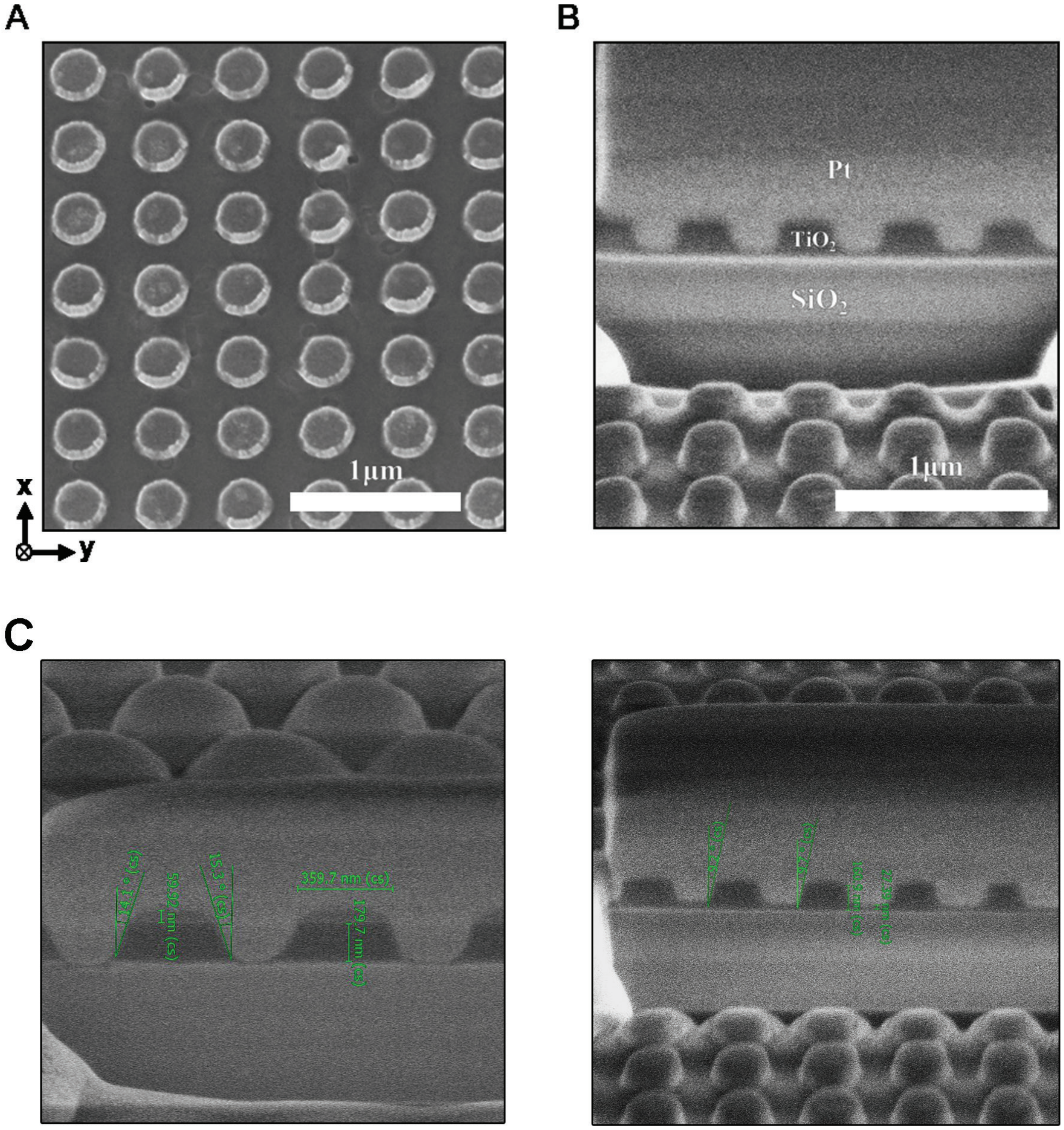

Figure S1. SEM image of the fabricated TiO_2 nanofrustums (90×90 μm^2). (A) Top view. (B) A cross-sectional view after sectioning the etched nanostructures using a focused ion beam. (C) Cross-sectional SEM images for two different samples. The obtained tapering angles are around 15˚ (left) and 9.2˚ (right), respectively.

2. Electromagnetic multipole expansion and radiation power

Table. S1 Primitive multipoles [1].

Electric dipole	$p_\alpha = \frac{1}{i\omega}\int d^3r\, J_\alpha$
Magnetic dipole	$m_\alpha = \frac{1}{2c}\int d^3r\, [\boldsymbol{r} \times \boldsymbol{J}]_\alpha$
Toroidal dipole	$T_\alpha = \frac{1}{10c}\int d^3r\, [(\boldsymbol{r}.\boldsymbol{J})r_\alpha - 2r^2 J_\alpha]$
Electric quadrupole	$Q^{(e)}_{\alpha,\beta} = \frac{1}{2i\omega}\int d^3r \left[r_\alpha J_\beta + r_\beta J_\alpha - \frac{2}{3}\delta_{\alpha,\beta}(\boldsymbol{r}.\boldsymbol{J})\right]$
Magnetic quadrupole	$Q^{(m)}_{\alpha,\beta} = \frac{1}{3c}\int d^3r\, [\boldsymbol{r} \times \boldsymbol{J}]_\alpha r_\beta + \{\alpha \leftrightarrow \beta\}$

where α, $\beta = x, y$.

Table. S2 Radiation power [2].

Electric dipole	$I_{p_\alpha} = \frac{2\omega^4}{3c^3}\lvert p_\alpha\rvert^2$
Magnetic dipole	$I_{m_\alpha} = \frac{2\omega^4}{3c^3}\lvert m_\alpha\rvert^2$
Toroidal dipole	$I_{T_\alpha} = \frac{2\omega^6}{3c^5}\lvert T_\alpha\rvert^2$
Electric quadrupole	$I_{Q^{(e)}_{\alpha,\beta}} = \frac{\omega^6}{5c^5}\sum \left\lvert Q^{(e)}_{\alpha,\beta}\right\rvert^2$
Magnetic quadrupole	$I_{Q^{(m)}_{\alpha,\beta}} = \frac{\omega^6}{20c^5}\sum \left\lvert Q^{(m)}_{\alpha,\beta}\right\rvert^2$

where α, $\beta = x, y$.

3. Simulated transmission of TiO_2 nanofrustum in free space

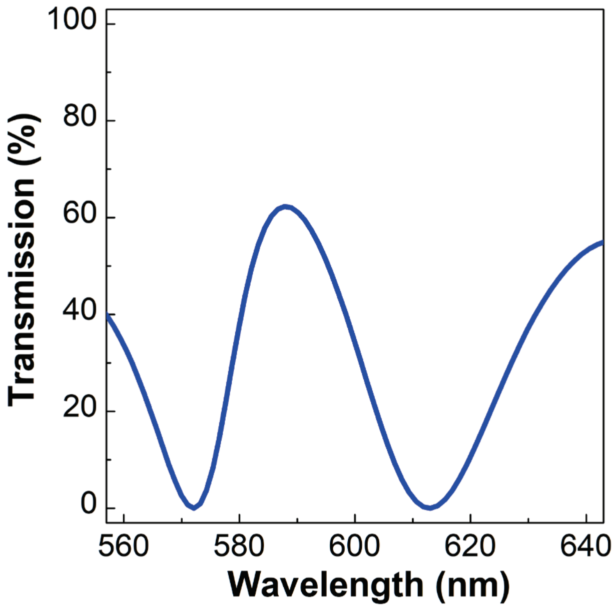

Figure S2. Simulated transmission of TiO_2 nanofrustums when the NR is absent. In comparison with the result shown in Fig. 1C, the resonant wavelengths perform a blue-shift when the NR is removed.

4. Simulated electric field distribution

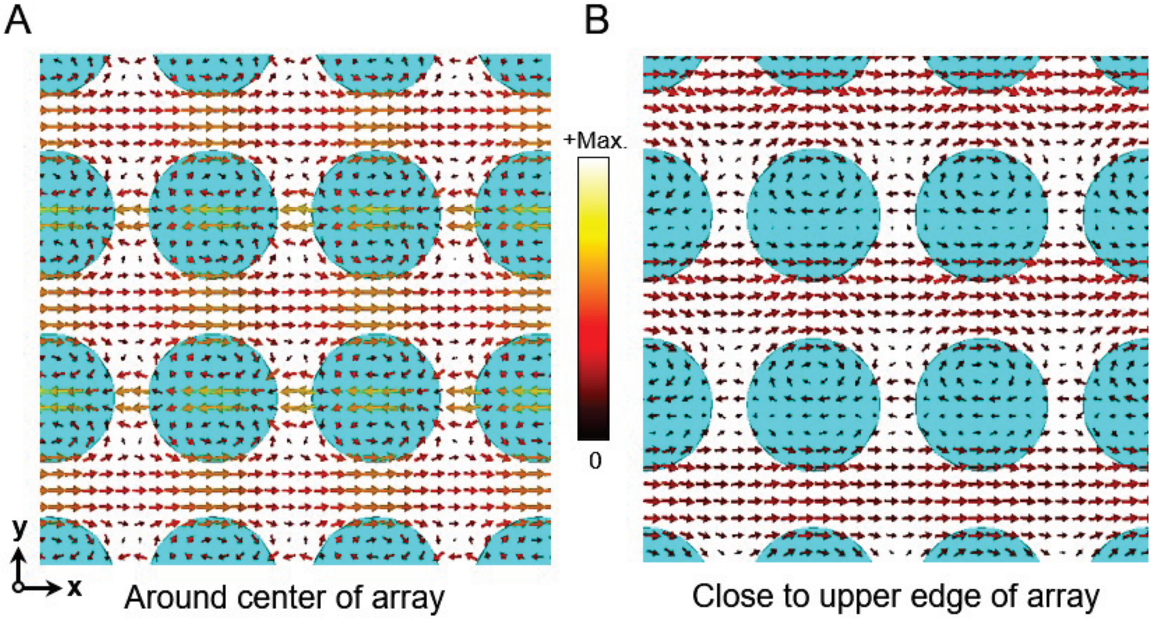

Figure S3. Simulated electric field distribution for the 68×68 metasurface array. (A) Field profile at the center of the array and (B) in the vicinity close to the edge of the array, both are numerically calculated at the electric resonance wavelength. In comparison with the field distribution at the edge, the electric field at the center of the array shows a relatively strong intensity and symmetric profile.

5. Ellipsometric measurement

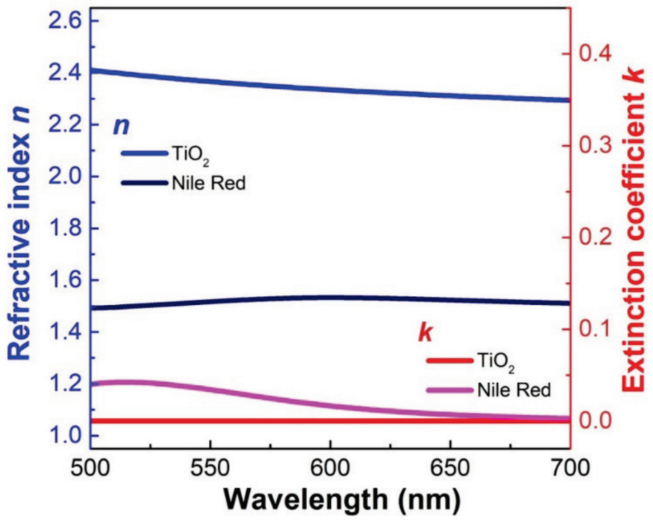

Figure S4. Measured optical refractive indices (*n* and *k*) for TiO_2 (blue and red solid curves) and NR+PMMA (dark blue and pink solid curves).

6. Optical setup for optical transmission and PL spectra measurement

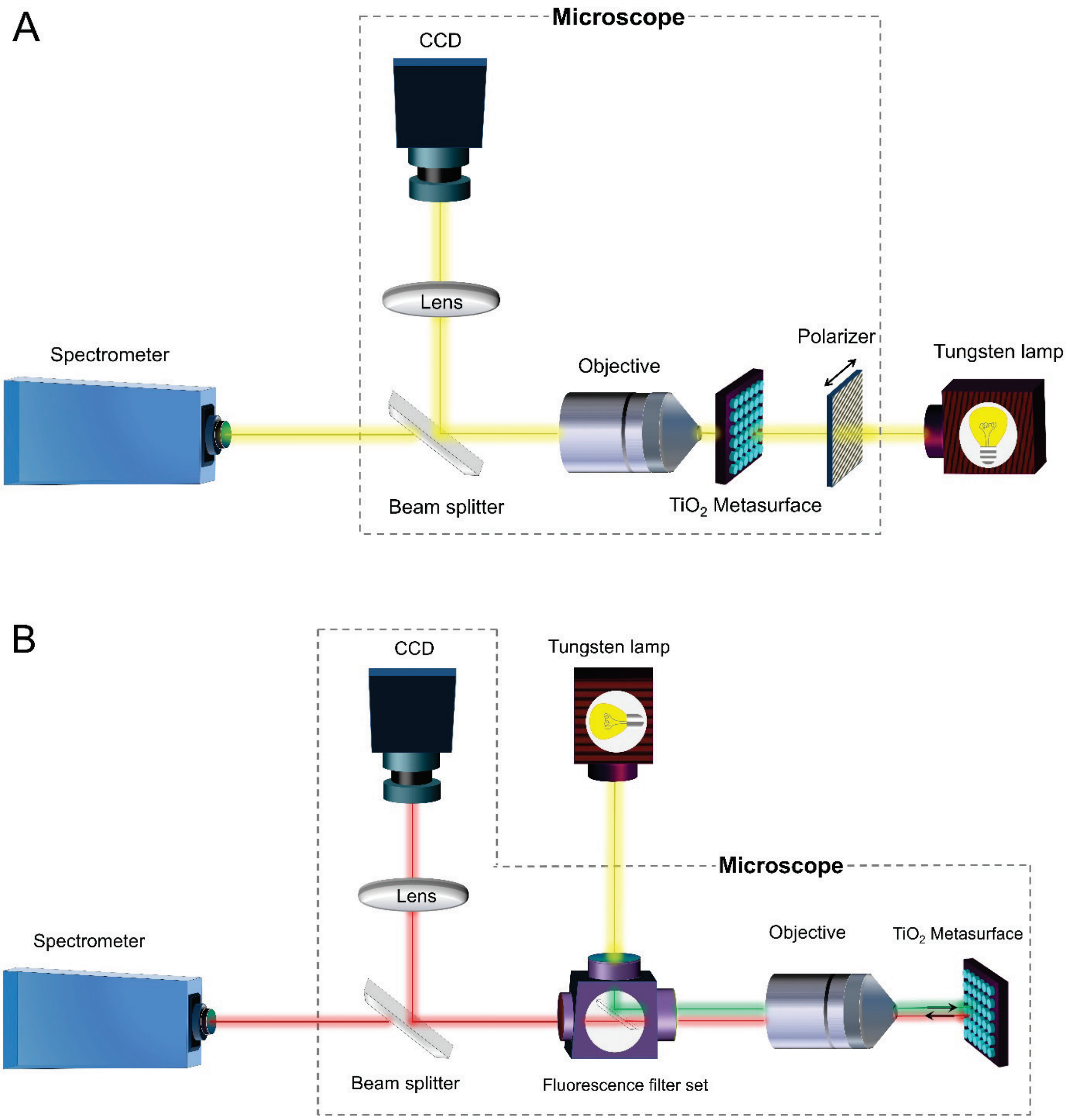

Figure S5. (A) Optical setup for the measurement of the transmission spectrum. The entire sample is illuminated with a tungsten lamp. A linear polarizer is inserted to determine the polarization state of incident light. The transmitted light is collected by a 50× objective (long working distance C plan objective: LCPLN50XIR) in a microscopic system equipped with a spectrometer (Andor Kymera 328i spectrometer system equipped with a visible CCD camera (DV401A-BVF) as the

detector). (B) The optical setup for PL measurement. A fluorescence filter set (U-FGW) is utilized to select the green light for excitation and to filter out the green light for detection.

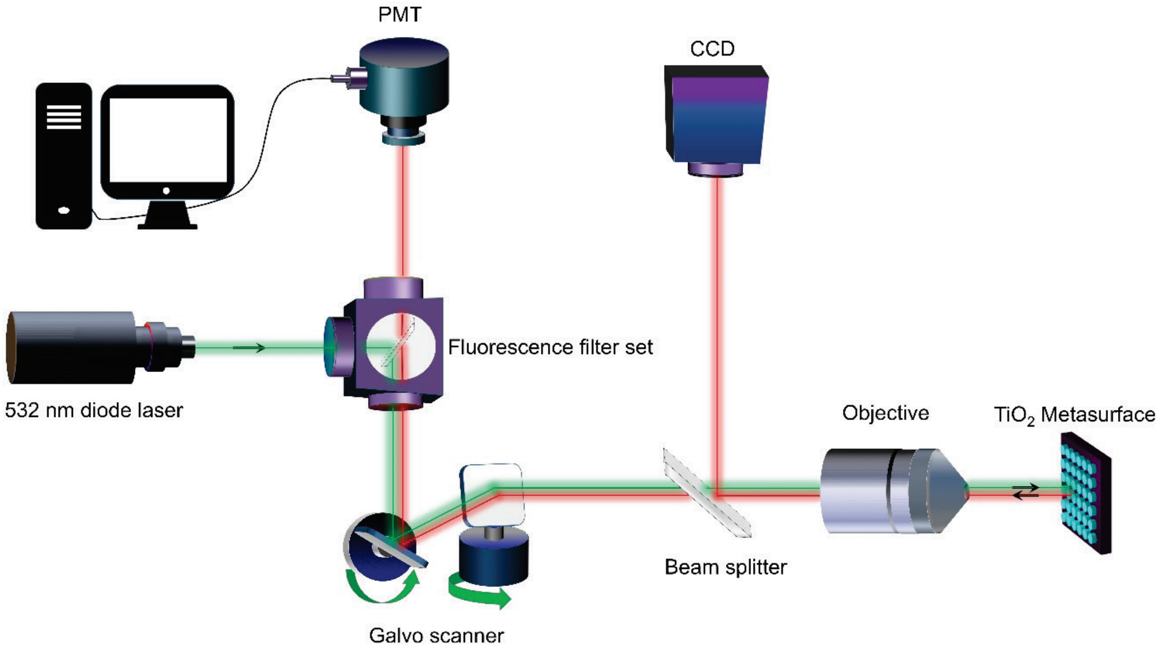

Figure S6. The spatial emission properties for PL mappings of the integrated TiO_2-NR metasurface samples are performed by using a home-built laser scanning confocal microscope (PSH 3G system, NI AD/DA/Counter card with PCI interface)[3] with a 532 nm diode laser excitation. An Olympus IX73, 20× (NA = 0.5) objective is used to focus the laser light onto the sample. While finding the region of interest with a charge-coupled device (CCD) DP80 camera (Olympus America, Central Valley, PA), the fluorescence imaging measurement collected by the same objective is focused onto the aperture of an UV-visible optical fiber (105 µm core) with an emission photomultiplier tube (PMT, 230 nm – 800 nm), which achieves a lateral resolution of ~532 nm (based on the Abbe's Law of limiting resolution) for fluorescence signals near the sample surface. The laser power at 0.35 mW through a 20× objective, with a power meter, is employed to excite the sample and filter for each PL map. A 532 nm dichroic mirror (LPD02-532RU-25) is used to filter out the excitation laser and to transmit the PL emission only.

7. The calculated Purcell factor of the TiO_2 metasurface

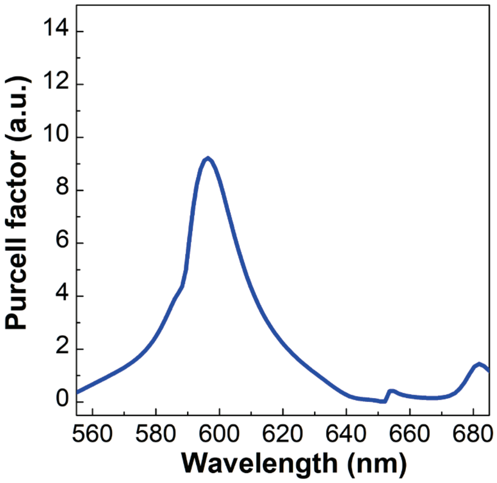

Figure S7. The calculated Purcell factor of the TiO_2 metasurface with an infinite array size. It is carried out by integrating the power flow through the unitcell from dipole emitters placing around the TiO_2 nanofrustums, and then dividing the value calculated in the model where the TiO_2 nanofrustums are absent.

References

[1] Savinov V, Fedotov VA, Zheludev NI. Toroidal dipolar excitation and macroscopic electromagnetic properties of metamaterials. Phys Rev B 2014;89:205112.

[2] Wu PC, Liao CY, Savinov V, et al. Optical anapole metamaterial. ACS Nano 2018;12:1920–7.

[3] Li H, Cheng HY, Chen WL, et al. Three dimensional characterization of GaN-based light emitting diode grown on patterned sapphire substrate by confocal Raman and photoluminescence spectromicroscopy. Sci Rep 2017;7:1–7.
